# The Structure-Activity Relationship of the Antioxidant Peptides from Natural Proteins

**DOI:** 10.3390/molecules21010072

**Published:** 2016-01-12

**Authors:** Tang-Bin Zou, Tai-Ping He, Hua-Bin Li, Huan-Wen Tang, En-Qin Xia

**Affiliations:** 1 Dongguan Key Laboratory of Environmental Medicine, School of Public Health, Guangdong Medical University, Dongguan 523808, China; zoutb@163.com (T.-B.Z.); taipinghe@163.com (T.-P.H.); 2 Guangdong Provincial Key Laboratory of Food, Nutrition and Health, Department of Nutrition, School of Public Health, Sun Yat-Sen University, Guangzhou 510080, China; lihuabin@mail.sysu.edu.cn

**Keywords:** structure-activity relationship, antioxidant peptides, proteins

## Abstract

Peptides derived from dietary proteins, have been reported to display significant antioxidant activity, which may exert notably beneficial effects in promoting human health and in food processing. Recently, much research has focused on the generation, separation, purification and identification of novel peptides from various protein sources. Some researchers have tried to discover the structural characteristics of antioxidant peptides in order to lessen or avoid the tedious and aimless work involving the ongoing generated peptide preparation schemes. This review aims to summarize the current knowledge on the relationship between the structural features of peptides and their antioxidant activities. The relationship between the structure of the precursor proteins and their abilities to release antioxidant fragments will also be summarized and inferred. The preparation methods and antioxidant capacity evaluation assays of peptides and a prediction scheme of quantitative structure–activity relationship (QSAR) will also be pointed out and discussed.

## 1. Introduction

Recently, bioactive peptides have received close scientific attention for their broad scope of bioactivities, mainly including antioxidation, antihypertensive, anticancer, and antimicrobial properties. Antioxidant peptides are especially prominent for their notable contributions to human health improvement through the prevention and treatment of non-communicable chronic degenerative diseases, such as cardiovascular and cerebrovascular diseases, cancer, rheumatoid arthritis or diabetes [1]. In addition, peptides with antioxidant properties exert effective metal ion (Fe^2+^/Cu^2+^) chelating activity and lipid peroxidation inhibitory capacity, which endows them with potential properties as food processing additives. The most attractive feature of peptides is their ability to display very few side effects in humans due to their natural sources.

It has been reported that bioactive peptides may originate from various natural proteins, such as cereals, legumes, milk, meat, egg, fish, and various marine organisms [2,3]. In fact, the search for novel bioactive peptides has increased considerably in the last few decades. Quite rich sources of proteins, including available food commodities, processing by-products and under-utilized resources, have been used to generate these peptides [2,3,4]. Bioactive peptides may be released during *in vivo* digestion, *in vitro* enzymatic hydrolysis or food processing steps, including fermentation, germination, and ripening [2,5]. Some results, including sources, evaluation methods, amino acid sequences and molecular weights of antioxidant peptides are listed in Table 1.

**Figure 1:**
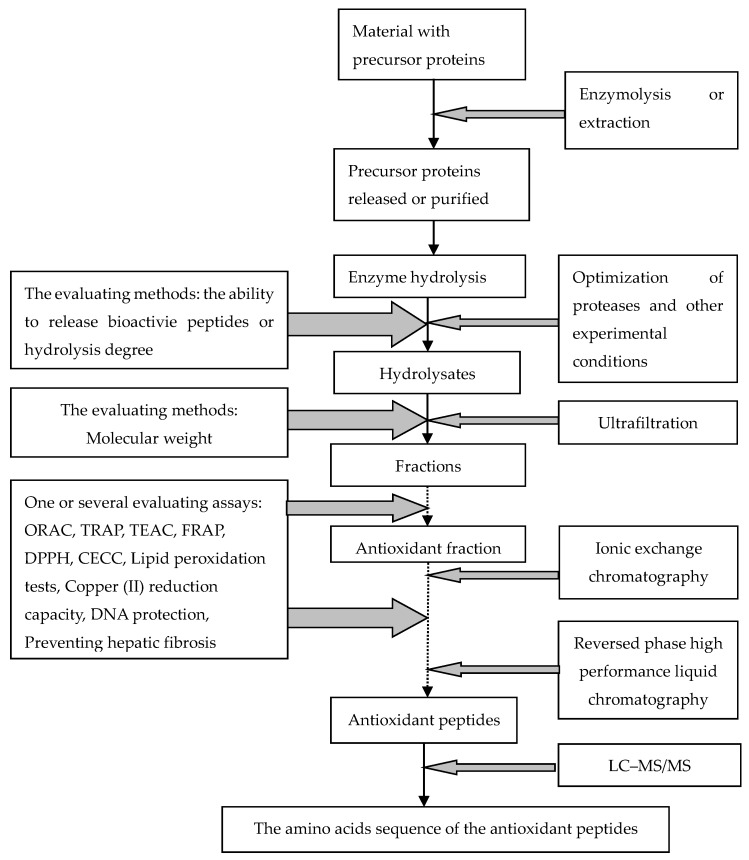
Preparation and identification of the novel antioxidant peptides from natural proteins. Note: the dotted line means that the process doesn’t always happen. The abbreviated indexes are: ORAC—Oxygen Radical Absorbance Capacity assay; TRAP—Total Radical trapping Antioxidant Parameter; TEAC—Trolox Equivalent Antioxidant Capacity; FRAP—Ferric ion-Reducing Antioxidant Power; DPPH—DiPhenyl-1–PicrylHydrazyl; CECC—Carnosine Equivalent iron Chelation Capacity; LC-MS/MS—liquid chromatography mass chromatography/mass chromatography.

Many efforts have been made to investigate and develop a series of methods for the enzymatic hydrolysis, isolation, purification and identification of novel peptides. Based on the recent literature, the main peptide preparation procedures are illustrated in Figure 1 [6,7,8]. No doubt, these works have made a huge contribution towards to exploration and identification of bioactive peptides. The databases on the sequences of peptides with antioxidant capacity is gradually increasing, however, it is still difficult for researchers to know beforehand the activities of the peptides resulting from certain enzyme hydrolyses. After the repeatitious and tedious work of the above procedures, the bioactivity of peptides obtained often shows conflicting results. How can we develop an efficient strategy for designing and producing novel natural-source peptides with strong antioxidant capacity? The knowledge of underlying mechanism of the antioxidant activity is very important. Nevertheless, any successful strategy must comprehensively clarify the structure-activity relationships of antioxidant peptides, as well as the relationships of the precursor protein which releases these antioxidant sequences.

This review was aimed at understanding and the prediction of the structure-activity relationship, and preparation of peptides with high activities more effectively. The major current knowledge and future perspectives in this field will also be discussed here.

**Table 1:** Sources, evaluation assays, and amino acid sequences of antioxidant peptides.

Source Protein	Assays	Sequence	Ref. *
Chicken egg white	ORAC	AEERYP, DEDTQAMP	[9]
Egg white	oxygen radical scavenging, DPPH radical scavenging.	DHTKE, MPDAHL, FFGFN	[10]
Rice residue protein	DPPH and ABTS radical scavenging, FRAP-Fe^3+^ reducing assay.	RPNYTDA, TSQLLSDQ, TRTGDPFF, NFHPQ	[11]
Grass carp (*Ctenopharyngodon idella*) skin	DPPH radical, hydroxyl radical, ABTS radical scavenging, Inhibiting lipid peroxidation.	PYSFK, GFGPEL, GGRP	[12]
Bluefin leatherjacket skin (*Navodon septentrionalis*)	DPPH, HO, O^2−^ radical scavenging.	GSGGL, GPGGFI, FIGP	[6]
Palm kernel cake proteins	DPPH radical scavenging, Metal chelating ability.	GIFE LPWRPATNVF	[13]
Blood clam *(Tegillarca granosa)* muscle	lipid peroxidation, radical scavenging activity.	WPP	[14]
Sweet potato	OH radical scavenging.	YYIVS	[15]
Croceine croaker (*Pseudosciaena crocea*) muscle	DPPH, superoxide, ABTS and hydroxyl radical scavenging, lipid peroxidation.	WLMSA, VLWEE, MILMR	[16]
Spotless smoothhound (*Mustelus griseus*) muscle	Hydroxyl, ABTS, superoxide radical scavenging.	GIISHR, ELLI, KFPE, GFVG, GAA	[17]
Bluefin leatherjacket (*Navodon septentrionalis*) heads	DPPH, hydroxyl, ABTS, superoxide radicals scavenging.	WEGPK, GPP, GVPLT	[18]
Hemp (*Cannabis sativa* L.) seed	DPPH radicals scavenging.	PSLPA, WVYY	[19]
Chickpea protein	DPPH radicals scavenging.	VGDI, DHG	[20]
Marine *Sepia brevimana* mantle	DPPH radicals scavenging, lipid peroxidation.	I/LNI/LCCN	[21]
*Sphyrna lewini* muscle	ABTS, DPPH radicals scavenging.	WDR, PYFNK	[22]
Tilapia (*Oreochromis niloticus*) gelatin	Hydroxyl radicals scavenging.	LSGYGP	[23]
Corn gluten meal	DPPH, ABTS, and hydroxyl radicals scavenging.	LLPF	[24]
Oyster *(Saccostrea cucullata)*	DPPH radicals scavenging, Inhibiting human colon carcinoma (HT-29) cell lines.	LANAK, PSLVGRPPVGKLTL, VKVLLEHPVL	[25]
Corp	Preventing hepatic fibrosis.	LLPF, FLPE	[26]

Note: ref. * -reference.

## 2. The Mechanism of the Antioxidation and the Evaluating Methods of Antioxidant Capacity

During the organism’s metabolism, reactive oxygen species (ROS) or free radicals are naturally produced by oxidation reactions through breathing. Living organisms have developed their own antioxidant defenses against excessive amounts of ROS. When their limited efficiencies cannot prevent all the oxidative damages related to environmental conditions (*i.e.*, UV light, non-equilibrated food and pollution), the excessive amounts of ROS finally cause an oxidative stress condition, which results in many non-communicable chronic diseases, such as diabetes, atherosclerosis, arthritis and cancer [27,28,29]. In addition, lipid peroxidation caused by ROS is one of the main causes of the degradation of lipids in food or cosmetic matrices [30]. Natural antioxidants have been found to possess the ability to effectively prevent the damage caused by ROS [31]. Therefore, there is a growing interest in exploring various antioxidant natural compounds, such as peptides derived from natural proteins.

Antioxidant capacity is used to evaluate the efficiency of natural antioxidants via two main chemical mechanisms. Free radical scavenging is the main role of antioxidants, which would be responsible for inhibiting electron migration (EM) and hydrogen atom transfer (HAT). In the literature [3,15], four antioxidant capacity assessment tests are commonly employed for the HAT-based reactions: the Oxygen Radical Absorbance Capacity (ORAC) assay; the Total Radical Trapping Antioxidant Parameter (TRAP) assay; the Crocin Bleaching Assay (CBA) and the Lipid Peroxidation Assay (LPA). For EM-based redox reactions, several antioxidant assays are commonly used such as Trolox Equivalent Antioxidant Capacity (TEAC); 2,2-diphenyl-1-picrylhydrazyl (DPPH) radical scavenging activity and Copper II Reduction Capacity (CRC) assay. Moreover, transient metal ion chelation is another contribution of natural antioxidants. The chemical mechanism involved is inhibition of the Fenton reaction, which is responsible for hydroxyl radical formation and subsequently leads to radical chain reactions. The transient metal ion chelating capacity of antioxidants is evaluated with the aid of two assays: EDTA Equivalent Iron Chelation Capacity (EECC) and Carnosine Equivalent Iron Chelation Capacity (CECC) [28]. In fact, when EDTA chelating capacity is too high compared to peptide chelating properties to investigate the iron chelating capacity of peptides, the use of carnosine as reference is advised. Some methods that have been applied in evaluating antioxidant properties of peptides are listed in Table 1.

The EC_50_ values, defined as the sample effective concentration required to achieve 50% activity was adopted by many researchers and allows the comparison of experimental results obtained from various studies. Some problems may occur that could cause deviation from the true results of antioxidant capacity of peptides during assessment. For example, in the ABTS assay, Tyr, Trp and their derivative peptides were strongly dependent on pH, while those of Cys and Cys-containing peptides were unaffected by pH. The former usually fail to reach equilibrium over the short incubation period of 6–10 min typically at low pH [32]. Furthermore, TEAC and FRAP are conducted at different pH values of 7.4 and 3.6, respectively. A significant distinction in structure of the same peptide may emerge due to the huge difference of pH conditions in the two reaction systems, especially for those peptides with acidic and non-neutral amino acid residues [33]. In addition, due to the different underlying mechanism between the evaluation assays, the values or variation trends obtained by different assays, such as ABTS radical scavenging assays, the metal chelating and hydroxyl radical scavenging assays, were found to be different, even for the same amino sequence and composition. This indicates that appropriately selecting the assays may provide a shortcut to accurately and effectively evaluating the antioxidant capacity, while deeply understanding the structure-activity relationships of the peptides may also give useful information for the selection of an appropriate assay.

## 3. The Relationship of Chemical Structure and the Antioxidant Ability of Peptides

The characteristic peptide chemical structures may be the main factors that influence their antioxidant activities. The precursor protein structures and their hydrolytic process may also affect peptide antioxidant activities.

### 3.1. Effect of the Structure of Precursor Proteins and the Hydrolytic Process on the Antioxidant Activities

By using a bioinformatic database of 745 sequences, Dziuba *et al.* predicted that the bioactive fragments released from the precursor proteins are usually located on protein surfaces with hydrophilic surroundings [5]. The structural properties of bioactive fragments were dominated by random coils (46%) and β-turns (30%) rather than β-sheets and α-helices. The prediction has been confirmed that β-sheet structures are found predominantly in β-lactoglobulin from bovine milk and proteins from legume seeds, which are possibly responsible for the on-release of bioactive peptides during gastrointestinal digestion [34].

Ahmed *et al.* reported that antioxidant peptides that could be released by the pepsin hydrolyzed whey and casein fractions of goat milk, but were not found in the hydrolysates of whole defatted goat milk using bacterial proteases [35]. Capriotti *et al.* compared the peptide generation capacity of protein extracted from soybean seeds with that obtained from untreated soy milk using *in vitro* gastrointestinal digestion. The numbers of potential bioactive peptides identified in extracted protein soybean seeds and soy milk, and untreated soy milk samples were 1173, 1422 and 1364, respectively. Obviously, there is significant gap in bioactive peptide generating capacity between soy seeds and its processed forms (soy milk) [36]. A reasonable explaination for the former is that it may be due to some changes in the molecular structure of precursor proteins during soy milk processing, while the impurities in unextracted soy milk proteins may affect peptide activities due to enzymolysis [36]. Furthermore, after *Amaranthus mantegazzianus* proteins were hydrolyzed with alcalase, the scavenging activities of the fractions increased, especially at a high degree of hydrolysis. One fraction called GlobPs with no initial activity exhibits the highest scavenging capacity after hydrolysis. However, the linoleic acid oxidation inhibitory capacity of GlobPs fraction that existed prior to enzymatic hydrolysis, due to the presence of naturally-occurring peptides/polypeptides, was partially lost after enzymatic hydrolysis [37].

These results suggest that the two objects of releasing certain fragments from proteins and maintaining its efficient domain must be taken into consideration during the exploition of and research on bioactive peptides. Comprehensively understanding the relationships between structure and the digestion of precursor proteins will shed light on the generation of novel antioxidant peptides.

### 3.2. The Relationship of Peptide Structure and Its Antioxidant Activity

Studies on the relationships between structure and antioxidant activity have been found in the literature, and the results showed that the antioxidant capacity of the peptides purified from proteins is closely related to some structural characteristic of the peptides, such as their molecular mass, amino acid compositions, sequences, and hydrophobicities [17,38].

#### 3.2.1. Molecular Weight

**Figure 2:**
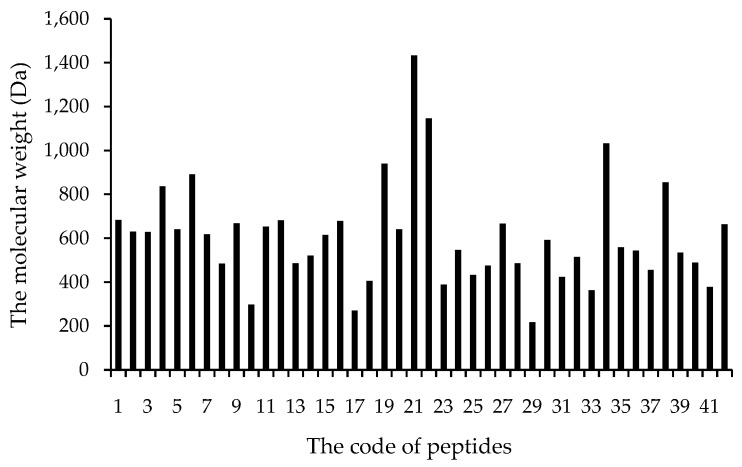
The molecular weight of 42 antioxidant peptides in Table 1.

The antioxidant peptides listed in Table 1 mainly have more than three amino acid residues, and are dominantly composed of 3–6 amino acids with molecular weights lower than 1000 Dalton (Da). The molecular weight distribution of those peptides is illustrated in Figure 2. The antioxidant peptides range from 400 to 650 Da, accounting for 70% of the 42 identified peptides (Figure 2). Several researchers have tried to show and explain the relationship between molecular weight and the antioxidant activity. The extraction of bioactive peptides from alcalase hydrolyzed residual materials from olive oil production yielded short chain peptides that exhibited significantly higher antioxidant capacities than their higher molecular weight counterparts [39]. Moreover, in the fractions of pinto bean protein hydrolysates using membrane ultrafiltration with molecular weight cutoffs of 100, 50, 30, 10 and 3 kDa, the peptide fraction <3 kDa exhibited the highest antioxidant activities [40]. The peptides below 1000 Da exhibit the best initial and sustained antioxidant activities in 2,2′-azinobis (3-ethylbenzothiszoline-6-sulphonic acid) diammonium salt (ABTS·+), hydroxyl radical scavenging and ORAC value tests [41]. In addition, one fraction of the alcalase hydrolysates of egg white protein, with M_W_ < 1 kDa possessed the strongest antioxidant ability compared with other ultrafiltration fractions [42]. The high activity against lipid peroxidation in a linoleic acid model system of BNH-P7 was due to the small size of peptides, because the more peptide sequences (282 peptides) will lead to a dilution effect of the peptides exhibiting hydroxyl radical scavenging activity [12]. Therefore, appropriately low molecular weight can exert a significant effect on the antioxidant activities of peptides.

#### 3.2.2. Amino Acid Composition

**Figure 3:**
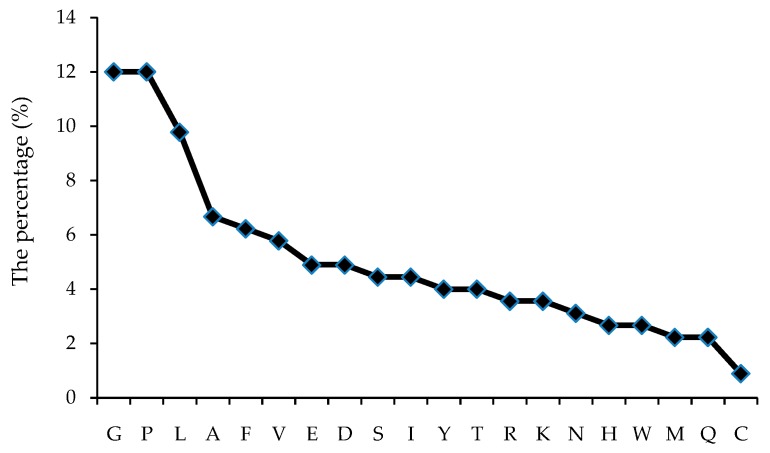
The percentage of each amino acid in the total AAs from 42 antioxidant peptides.

It was found that a total of 20 amino acids in the human body exist in the 42 antioxidant peptides (Table 1). To track some preferential amino acids for their antioxidant activities, the number of each component amino acid in the 42 peptides in Table 1 were analyzed and the result is illustrated in Figure 3. The differences in the percentages of amino acids indicate the unequal distribution of each amino acid in peptides. A larger number of some amino acids, such as Gly (G), Pro (P), Leu (L), account for 33.7% of total amino acids, and Ala (A), Tyr (F) and Val (V), 18.7%. Cys (S) were found as the least abundant amino acid following Met (M) and Gln (Q), which accounted for 4.9% of the number of amino acids in those antioxidant peptides.

The effect of the amino acid composition of the peptides on antioxidant activity has been stressed in the literature. A high proportion of hydrophobic amino acids has been reported in peptides with high antioxidant activity, compared to other hydrophilic amino acids, which is considered as the key factor in peptide ability to scavenge radicals. For instance, the nanofiltration fraction (1–4 kDa) from tuna dark muscle by-product showed the highest superoxide radical and reducing power activities, which contained antioxidant amino acids such as Tyr, Phe, Pro, Ala, His, and Leu, which accounted for 30.3% of the total amino acids [43]. Furthermore, in three enzymolyzing systems, the highest antioxidant activity peptides from marine *Sepia brevimana* mantle was present in pepsin hydrolysates, which may be due to increased exposure of internal hydrophobic amino acids during hydrolysis of the peptides in the smaller fractions compared to other hydrolyzing enzymes [21]. The antioxidant activity followed a sequence order of Pro-Tyr-Ser-Phe-Lys > Gly-Phe-Gly-Pro-Glu-Leu > Val-Gly-Gly-Arg-Pro [12] for DPPH, OH radical and ABTS assays, while the order Trp-Pro-Pro > Gln-Pro was for hydroxyl radical scavengers, thus confirming the inference above [14]. The mechanism of action may be that the antioxidant peptides could smoothly enter into target organs through hydrophobic interactions with membrane lipid bilayers by the aid of their hydrophobicity, where they are able to exert significant capacity of scavenging radicals [30].

**Figure 4:**
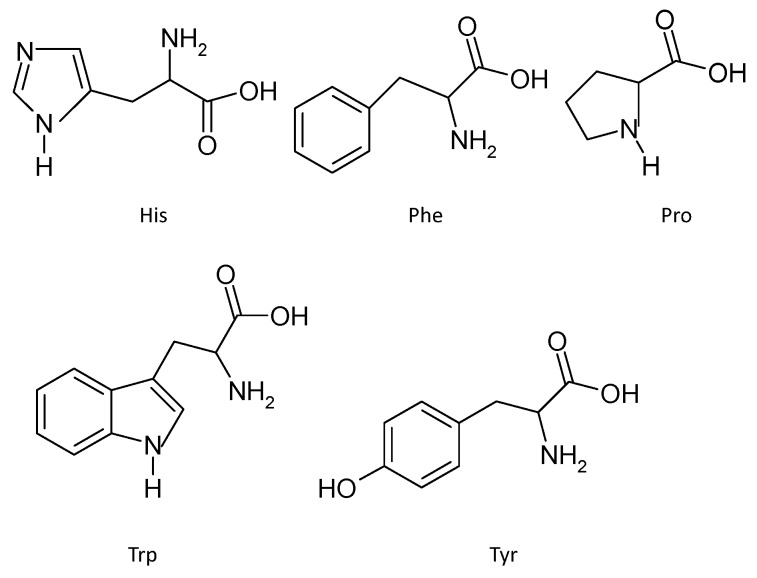
The chemical structures of five antioxidant amino acids with ring.

Hydrophobic amino acids tested frequently include His, Trp, Phe, Pro, Gly, lys, Ile and Val. His residues are credited with strong radical scavenging activity in oxidative reactions, especially for enzyme-catalyzed reactions, due to the presence of an imidazole ring as an important proton donor [3]. Torres-Fuentes *et al.* obtained most antioxidant peptides from chickpea proteins, which commonly contains His residures [44]. Another study showed the antioxidant capacity of peptides could be enhanced notably by the presence of three aromatic amino acids (Trp, Tyr and Pre) [19]. In addition, the indole and pyrrolidine ring in Trp and Pro, respectively, could also serve as hydrogen donors via their hydroxyl groups, thus acting as hydroxyl radical scavengers. Furthermore, Mendis *et al.* suggested that higher radical scavenging activity was observed when peptides contained two Phe and two His residues [45]. The other amino acids, *i.e.*, Gly, Lys, Ile and Val, may be responsible for forming a favorable hydrophobic micro-environment for peptide molecules. A comparison of seven peptide residues’ (Leu-His-Tyr, Leu-Ala-Arg-Leu, Gly-Gly-Glu, Gly-Ala-His, Gly-Ala-Trp-Ala, Pro-His-Tyr-Leu and Gly-Ala-Leu-Ala-Ala-His) scavenging activities: showed that the first peptide (Leu-His-Tyr) displayed the highest DPPH radical-scavenging activity (63% ± 1.57%; at 150 μg/mL), which may confirm the foregoing theories [46]. According to Tian *et al.* Cys was predicted by a QSAR model to be the most active antioxidant amino acid when found in tripeptides, yet there are few existing peptides reported whose activities may be due to the formation of strong disulfide bonds in the precursor molecules [33]. The chemical structures of His, Trp, Phe, Tyr and Pro are displayed in Figure 4.

In fact, excessive hydrophobic amino acids means low solubility, which induces the low ABTS and high hydroxyl radical scavenging activity. For instance, when compared with Gly-Ser-Gly-Gly-Leu and Gly-Pro-Gly-Gly-Phe-Ile, Phe-Ile-Gly-Pro exhibited the highest DPPH- (EC_50_ 242 μM), HO- (EC_50_ 150 μM), and O_2_-(EC_50_ 640 μM) scavenging activities and effectively inhibited autooxidation in a linoleic acid model system [6].

The negatively charged acidic amino acids, *i.e.*, Glu and Asp, display free radical quenching activity due to the presence of excess electrons. For instance, the rapeseed peptides (RPs) prepared by solid state fermentation with *Bacillus subtilis* display a concentration-dependent effect on antioxidant activities. The dominant amino acids in the peptides were glutamic acid (19.5%), lysine (7.6%) and proline (7.3%), with least amounts of serine (1.5%), tryptophan (1.3%) and cysteine (0.5%). The peptides showed high activities of scavenging free radicals, reducing power, and inhibition of lipid peroxidation, but low ferrous ion-chelating activity [47].

According to the results reported by of Chen *et al.*, (in press) and Tironi *et al.* the antioxidant capacity of peptides can decrease or completely lost after hydrolysis or be changed into free amino acids [37,48]. Chen *et al.* found that defatted walnut meal hydrolysates exhibited relatively strong hydroxyl scavenging and oxygen radical absorbance capacities, protective effect on H_2_O_2_-injured PC12 cells [48]. The mixture with the same components of amino acids could not offer the same protection effect suggesting that besides the concentration of antioxidant amino acids, the relative spatial structure of the amino acids in the peptide sequence might also play an important role in its antioxidant activity [49].

#### 3.2.3. Amino Acids Sequence 

The interactions between amino acids may be an important factor for the antioxidant activity. Besides the hydrophobicity of peptides with high antioxidant activity, the amphiphilic nature of peptides also seems to enhance the radical-scavenging activities by increasing peptide solubility while facilitating interaction and proton exchanges with radical species. For example, when myofibrillar protein from patin was hydrolysed using papain, the FVNQPYLLYSVHMK peptide exhibited the highest antioxidant activity. In the sequence of the peptide, the presences of hydrophobic amino acids (Leu, Val and Phe), hydrophilic and basic amino acids (His, Pro and Lys), and aromatic amino acids (Phe and Tyr) in the peptide sequences are believed to contribute to its overall high antioxidant activity [50]. In addition, the antioxidant activity of two peptides, namely, Gly-Pro-Pro and Trp-Glu-Gly-Pro-Lys were investigated. The results showed that Gly-Pro-Pro exhibited the highest scavenging activity on DPPH radicals (EC_50_ 6310 μM), hydroxyl radicals (EC_50_ 7700 μM), and ABTS radicals (EC 8100 μM), whereas Trp-Glu-Gly-Pro-Lys could not effectively inhibit the peroxidation of linoleic acid [18]. The mechanism behind these results may stem from the inhibiting lipid peroxidation assays being conducted in the presence of lipids (e.g., FTC assay) or an emulsion, which would help the amphiphilic properties of the antioxidants enter into the lipid system so that they could react with the free radicals.

Bamdad and coworkers have investigated the interaction effects among the amino acids in the peptide sequences on the antioxidant capacity in detail [51]. Six peptides were synthetized according the structural motif of the most potent antioxidant peptides derived from barley protein, which contained pentapeptide Gln-Pro-Tyr-Pro-Gln with appended Gln and Pro residues. The contribution of vicinal residues to their antioxidant capacity was investigated by using repetitive sequences of Gln, Pro and Tyr. The results showed that the peptides displayed a significant positive role in stabilizing the peptide radicals in the free radical scavenging assays, as well as in effectively inactivating lipid hydroperoxides, intracellular reactive oxygen species, and inhibition of amyloid fibril formation in a cellular model. The carboxyl of Glu located next to Tyr could induce the release of the hydrogen atom of the phenolic hydroxyl in Tyr, enhancing the antioxidant activity. The results confirm that combined effect of the Gln-Pro and Pro-Tyr pairs and appending Gln and Pro on antioxidant capacity of peptides is efficient [51].

In addition, Trp in the sequence is another good example of influence on the antioxidant activity of neighboring amino acid residues. Trp has been determined to influence high antioxidant activity, and shows various activities in different sequences. The reason may be the special capacity of the indole ring of Trp to serve as an active hydrogen donor of the indole radical, and the stability it provides to neighboring amino acid residues with different physicochemical properties, which influence the antioxidant activity of the total peptides [33]. The sulfhydryl (SH) group in Cys is a hydrogen donor which comes from the SH group or the loss of an electron from its sulfur atom. The Trp residues with an aromatic ring structure have been reported to donate a proton to electron deficient radicals. Therefore, Cys and Trp would make a significant contribution to the antioxidant activities of certain peptides, when they are present in the same peptide chain.

The characteristic structure of the amino acids linked to the metal chelating ability of peptides was investigated by Canabady-Rochelle *et al.* [52]. According to their results, there is a excellent linear relationship between His-peptides and its metal chelating ability with *R*^2^ values amounting to 0.9 [52]. The iron chelating capacity was inferred involving the -NH group of the imidazole ring of the histidine residue, especially when His is located at the C-terminal extremity, which can chelate the Fe^2+^ ion through coordination bond. Moreover, as being located at the *N*-terminal of the peptides, the β-Ala can drastically enhance the chelation capacity of His. However, the effect does not happen to α-Ala, which cannot form two hydrogen bonds as easily as β-Ala. Moreover, the single indole ring of the Trp residue was not involved in the iron chelation mechanism. However, the best half maximal inhibitory concentration values measured using metal chelating activity were shown by LPWRPATNVF, AWFS and YGIKVGYAIP to be 0.001 μM, 0.002 μM and 0.087 μM, respectively [13]. The metal chelating activity of the three peptides may be related to the combined effects of the indole, benzene and phenol rings existing in Trp, Phe and Tyr, respectively. Furthermore, the values of the chelating capacity indicate that the synergetic effect of two phenol rings in YGIKVGYAIP may be considerable poorer than that between an indole ring and a benzene ring in LPWRPATNVF and AWFS. Trp and Phe will even improve the synergistic action due to the larger space provided for metal ions. Therefore, the interaction of amino acids and the relative location in the sequence can significantly influence the antioxidant capacity of peptides.

A sequence of hydrophobic amino acid residues like Val or Leu at the *N*-terminus containing Pro, His or Tyr, is characteristic of antioxidant peptides. In the sequence, Pro tends to interrupt and change the secondary structure of: the peptide molecule, which may increase the availability of the amino acid residues of the peptide sequences to act as antioxidants [53]. Leu and the Ser-Leu/Th r-Leu/Pro-Leu motifs in their sequences, particularly at the N- and C-terminus, seem to contribute to their superior scavenging activities [54]. According to the results obtained by Li and Li, antioxidant activity is correlated with structure related to the electron properties, hydrophobicity, stericity, and hydrogen bonding properties of amino acids for tripeptides [55]. The electron properties of amino acid residues are very important, and bulky hydrophobicity at the C-terminal is also closely related to the antioxidant activity. The results suggest that the electronic, hydrogen-bonding properties and location of the amino acids, along with the steric properties of the amino acid residues at the C- and N-termini may be the root of the antioxidant activity of the whole peptides.

#### 3.2.4. Secondary Structure

Taking the secondary structure into consideration, Raghava *et al.* found by bioinformatics that 58 out of 77 bioactive peptides (accounting for 75% of total peptides) contained at least one β-turn, followed by helices (60%), and β-strands were present in just 13% of the total peptides studied [56]. Based on the conformational prediction and fourier transformed infrared spectroscopy, a β-sheet structure exists in HLFGPPGKKDPV (MW: 1291.51 Da), derived from fertilized hen eggs [8]. The two thiol-bearing peptide of apolipoprotein (apo) A-IMilano (R173C) possesses novel antioxidant properties on phospholipid surfaces in a dose-dependent manner. The contextual constraints of the amphipathic α-helices play the key role in the antioxidation [57]. α-Helices and β-sheet secondary structures exist in the structure of antioxidant peptides, but results verifying a role of the secondary structure on the activity are quite scarce. According to the previous sections, the peptides with significant antioxidant capacity are usually short chains with 4–6 residues. Therefore, the secondary structure may be a minor factor for the antioxidant peptides due to their low molecular weight.

#### 3.2.5. Antioxidant Peptides’ Stability and Their Synergistic Effects

Peptides exert their antioxidant activity as an integrated molecule. However, they are probably digested into amino acids by endogenous disgestive enzymes before reaching the target organs. It is important for peptides to resist proteolysis by gastrointestinal, brush border and serum peptidases, in humans. The antioxidant peptides from whole thermolysin extract was found to resist gastrointestinal disgestion with little change in structures and antioxidant activity [4]. Some special molecular structures remaining intact have been found after passage through the gastrointestinal tract. Chen and the coworkers reported that peptides above 3000 Da were more inclined to be cut up during gastric-intestinal digestion than those below 3000 Da [41]. The antioxidant activity of the product mainly consisted of five oligopeptides (molecular weight: 202.1, 294.1, 382.1, 426.3, and 514.4 Da) from duck egg white kept stable after *in vitro* digestive simulation [38]. Moreover, *in vitro* gastrointestinal digestion has been applied to produce hydrophobic peptides of 0.75 kDa with soybean lipoxygenase [58]. Obviously, low molecular weight (<1 kDa) was the useful characteristic for the antioxidant activity of peptides. On the other hand, like the precusor proteins, some compact structural features may resist the *in vitro* gastrointestinal digestion, such as the β-sheet structures or disulfide-rich ones with head-to-tail cyclic peptides like lunasin. According to research results, lunasin can exert its bioefficacy by maintaining its native structure during heating (100 °C, 10 min), protease degradation or gastrointestinal disgestion [8,59,60].

In addition, significantly enhanced antioxidant activity after fractionation has also found [38,61]. However, a decreasing trend of antioxidant capacity of hydrolysates after fractionation has been found [19]. The results suggest a considerably wide presence of synergistic influence of antioxidant activity among peptides [4,7,11]. Understanding the underlying structural mechanism of the synergistic effect will be useful for exploring novel antioxidant peptides.

## 4. Systematic Schemes to Predict the Antioxidative Activity of Peptides

The bioactivity of peptides may result from their chemical structure. Recently, a quantitative structure-activity relationship (QSAR) scheme as an application of chemometric and bioinformatics methods has been used [62]. It has been used extensively in this context in the fields of medical science and toxicology [33,55,63]. The characteristic chemical structure of antioxidant peptides can be predicted by a model expressed as a mathematical expression for the relationship between structure and activity. It may be a useful tool to evaluate food proteins as potential precursors of bioactive peptides and to predict the possible release of bioactive peptides from various proteins using proteolytic enzymes with different specificity. QSAR models involving antioxidant peptides have been established [33,63]. Tian and coworkers reported that by QSAR models with the FRAD assay, some structural characteristics, including electron and hydrogen-bonding properties of the amino acids, the steric properties of the amino acid residues at the C- and *N*-termini and the neighboring amino acids of the Cys or Trp units are predicted to play an important role in the antioxidant activities of the tripeptides from β-lactoglobulin [33]. Li and Li have also successfully predicted the relationship of the C-terminal and *N*-terminal regions and antioxidant potency using QSAR models with three databases, namely, Trolox-equivalent antioxidant capacity (TEAC), oxygen radical absorption capacity (ORAC), and superoxide radical (SOR) [55]. For predicting antioxidant activity, the properties of amino acids at the C-terminal regions were found more important than those at the N-terminal regions. The electronic donating ability is found to significantly influence the antioxidant activity in the three free radical systems. Furthermore, their synergistic effects are displayed notably at each position. Bulky hydrophobic amino acids at the C-terminal were related to the antioxidant activity of peptides in the three free radical systems. Some TEAC databases show the probable influence of stericity in the N-terminal segment (N2, N3) and the activity exists [53].

Bioinformatics, as well as QSAR models, can provide insight at the molecular level of specific peptide sequences. Most limitations on the application of QSAR in exploring antioxidant peptides from protein hydrolysates cannot be neglected. The distinguishing features for using the bioinformatics methods is heavily dependent on the relative data. The results reported in the field mainly focus on tripeptides for their simple structures. Since only peptides with the same number of amino acids were used to illustrate the QSAR modeling, the knowledge of the structure-activity relationships of peptides longer than four amino acids is far from being accessible as the process becomes more complicated with longer peptides. Further investigations are often limited by the high cost of chemical synthesis of longer peptides, which may involve cross-validation tests to objectively evaluate the anticipated accuracy of the predictor. The data in the literature cannot be fully used due to the defects of evaluation assays, nevertheless, it will be a useful tool for initiative and systemic research on antioxidant peptides hydrolyzed from proteins [64].

## 5. Conclusions

Antioxidant peptides have been widely studied for their significant prevention and improvement of the conditions of non-communicable chronic degenerative diseases and beneficial effects on food processing. Aided by several evaluation assays, the structure-activity relationships of the peptides obtained from different proteins have been investigated. The various characteristics of chemical structure, namely, small molecular weight, amino acids (His, Trp, Phe, Pro, Gly, lys, Ile and Val) with hydrophobicity, indole/imidazole/pyrrolidine ring, along with the steric structure at the C- and N-termini and the neighboring amino acids of some residues, all show an influence on the antioxidant activity of peptides. Among them, the composition and the sequence of amino acids have the most impact on the antioxidant activity. In addition, the bioinformatics methodology of QSAR shows significant advantages for generating novel antioxidant peptides, nevertheless, its development is still in its infancy. The ongoing tedious, cost- and time consuming work of exploring the novel antioxidant peptides can be changed into a simple, systematic and designed procedure aided by the rich knowledge of the structure-activity relationship. Combining the application of bioinformatics methodology with the exploration of novel peptides could significantly promote the development in this field of specialization.

## References

[1] Montoya-Rodriguez A., de Mejia E.G. (2015). Pure peptides from amaranth (*Amaranthus hypochondriacus*) proteins inhibit LOX-1 receptor and cellular markers associated with atherosclerosis development *in vitro*. Food Res. Int..

[2] Moller N.P., Scholz-Ahrens K.E., Roos N., Schrezenmeir J. (2008). Bioactive peptides and proteins from foods: Indication for health effects. Eur. J. Nutr..

[3] Samaranayaka A.G.P., Li-Chan E.C.Y. (2011). Food-derived peptidic antioxidants: A review of their production, assessment, and potential applications. J. Funct. Foods.

[4] Vásquez-Villanueva R., Marina M.L.M., Garcia C. (2015). Identification by hydrophilic interaction and reversed-phase liquid chromatography-tandem mass spectrometry of peptides with antioxidant capacity in food residues. J. Chromatogr. A.

[5] Dziuba J., Niklewicz M., Iwaniak I., Darewicz M., Minkiewicz P. (2004). Bioinformatic-aided prediction for release possibilities of bioactive peptides from plant proteins. Acta. Aliment..

[6] Chi C.F., Wang B., Hu F.Y., Wang Y.M., Zhang B., Deng S.J., Wu C.W. (2015). Purification and identification of three novel antioxidant peptides from protein hydrolysate of bluefin leatherjacket (*Navodon septentrionalis*) skin. Food Res. Int..

[7] Song R., Wei R.B., Ruan G.Q., Luo H.Y. (2015). Isolation and identification of antioxidant peptides from peptic hydrolysates of half-fin anchovy (*Setipinna taty*). LWT-Food Sci. Technol..

[8] Duan X., Ocen D., Wu F.F., Li M., Yang N., Xu J., Chen H.Y., Huang L.Q., Jin Z.Y., Xu X.M. (2014). Purification and characterization of a natural antioxidant peptide from fertilized eggs. Food Res. Int..

[9] Nimalaratne C., Bandara N., Wu J.P. (2015). Purification and characterization of antioxidant peptides from enzymatically hydrolyzed chicken egg white. Food Chem..

[10] Liu J.B., Jin Y., Lin S.Y., Jones G.S., Chen F. (2015). Purification and identification of novel antioxidant peptides from egg white protein and their antioxidant activities. Food Chem..

[11] Yan Q.J., Huang L.H., Sun Q., Jiang Z.Q., Wu X. (2015). Isolation, identification and synthesis of four novel antioxidant peptides from rice residue protein hydrolyzed by multiple proteases. Food Chem..

[12] Cai L.Y., Wu X.S., Zhang Y.H., Li X.X., Ma S., Li J.R. (2015). Purification and characterization of three antioxidant peptides from protein hydrolysate of grass carp (*Ctenopharyngodon idella*) skin. J. Funct. Foods.

[13] Zarei M., Ebrahimpour A., Abdul-Hamid A., Anwar F., Bakar F.A., Philip R., Saari N. (2014). Identification and characterization of papain-generated antioxidant peptides from palm kernel cake proteins. Food Res. Int..

[14] Chi C.F., Hu F.Y., Wang B., Li T., Ding G.F. (2015). Antioxidant and anticancer peptides from the protein hydrolysate of blood clam (*Tegillarca granosa*) muscle. J. Funct. Foods.

[15] Zhang M., Mu T.H., Sun M.J. (2014). Purification and identification of antioxidant peptides from sweet potato protein hydrolysates by Alcalase. J. Funct. Foods.

[16] Chi C.F., Hu F.Y., Wang B., Ren X.J., Deng S.J., Wu C.W. (2015). Purification and characterization of three antioxidant peptides from protein hydrolyzate of croceine croaker (*Pseudosciaena crocea*) muscle. Food Chem..

[17] Wang B., Gong Y.D., Li Z.R., Yu D., Chi C.F., Ma J.Y. (2014). Isolation and characterisation of five novel antioxidant peptides from ethanol-soluble proteins hydrolysate of spotless smoothhound (*Mustelus griseus*) muscle. J. Funct. Foods.

[18] Chi C.F., Wang B., Wang Y.M., Zhang B., Deng S.J. (2015). Isolation and characterization of three antioxidant peptides from protein hydrolysate of bluefin leatherjacket (*Navodon septentrionalis*) heads. J. Funct. Foods.

[19] Girgih A.T., He R., Malomo S., Offengenden M., Wu J.P., Aluko R.E. (2014). Structural and functional characterization of hemp seed (*Cannabis sativa* L.) protein-derived antioxidant and antihypertensive peptides. J. Funct. Foods.

[20] Ghribi A.M., Sila A., Przybylski R., Nedjar-Arroume N., Makhlouf I., Blecker C., Attia H., Dhulster P., Bougatef A., Besbes S. (2015). Purification and identification of novel antioxidant peptides from enzymatic hydrolysate of chickpea (*Cicer arietinum* L.) protein concentrate. J. Funct. Foods.

[21] Sudhakar S., Nazeer R.A. (2015). Preparation of potent antioxidant peptide from edible part of shortclub cuttlefish against radical mediated lipid and DNA damage. LWT-Food Sci. Technol..

[22] Wang B., Li Z.R., Chi C.F., Zhang Q.H., Luo H.Y. (2012). Preparation and evaluation of antioxidant peptides from ethanol-soluble proteins hydrolysate of *Sphyrna lewini* muscle. Peptides.

[23] Sun L.P., Zhang Y.F., Zhuang Y.L. (2013). Antiphotoaging effect and purification of an antioxidant peptide from tilapia (*Oreochromis niloticus*) gelatin peptides. J. Funct. Foods.

[24] Zhuang H., Tang N., Yuan Y. (2013). Purification and identification of antioxidant peptides from corn gluten meal. J. Funct. Food.

[25] Umayaparvathi S., Meenakshi S., Vimalraj V., Arumugam M., Sivagami G., Balasubramanian T. (2014). Antioxidant activity and anticancer effect of bioactive peptide from enzymatic hydrolysate of oyster (*Saccostrea cucullata*). Biomed. Prev. Nutr..

[26] Wang C., He H., Zhang J.L., Li X., Ma Z.L. (2015). High performance liquid chromatography (HPLC) fingerprints and primary structure identification of corn peptides by HPLC-diode array detection and HPLC-electrospray ionization tandem mass spectrometry. J. Food Drug Anal..

[27] Borges R.S., Castle S.L. (2015). The antioxidant properties of salicylate derivatives: A possible new mechanism of anti-inflammatory activity. Bioorgan. Med. Chem. Lett..

[28] Huang D., Ou B., Prior R.L. (2005). The chemistry behind antioxidant capacity assay. J. Agric. Food Chem..

[29] Mirzaei M., Mirdamadi S., Ehsani M.R., Aminlari M., Hosseini E. (2015). Purification and identification of antioxidant and ACE-inhibitory peptide from Saccharomyces cerevisiae protein hydrolysate. J. Funct. Foods.

[30] Pouzo L.B., Descalzo A.M., Zaritzky N.E., Rossetti L., Pavan E. (2016). Antioxidant status, lipid and color stability of aged beef from grazing steers supplemented with corn grain and increasing levels of flaxseed. Meat Sci..

[31] Shi Y., Kovacs-Nolan J., Jiang B., Tsao R., Mine Y. (2014). Peptides derived from eggshell membrane improve antioxidant enzyme activity and glutathione synthesis against oxidative damage in Caco-2 cells. J. Funct. Foods.

[32] Zheng L., Zhao M., Xiao C., Zhao Q., Su G. (2016). Practical problems when using ABTS assay to assess the radical-scavenging activity of peptides: Importance of controlling reaction pH and time. Food Chem..

[33] Tian M., Fang B., Jiang L., Guo H., Cui J.Y. (2015). Structure-activity relationship of a series of antioxidant tripeptides derived from β-Lactoglobulin using QSAR modeling. Dairy Sci. Technol..

[34] Carbonaro M., Nardini M., Maselli P., Nucara A. (2015). Chemico-physical and nutritional properties of traditional legumes (lentil, *Lens culinaris* L., and grass pea, *Lathyrus sativus* L.) from organic agriculture: An explorative study. Org. Agric..

[35] Ahmed A.S., El-Bassiony T., Elmalt L.M., Ibrahim H.R. (2015). Identification of potent antioxidant bioactive peptides from goat milk proteins. Food Res. Int..

[36] Capriotti A.L., Caruso G., Cavaliere C., Samperi R., Ventura S., Chiozzi R.Z., Laganà A. (2015). Identification of potential bioactive peptides generated by simulated gastrointestinal digestion of soybean seeds and soy milk proteins. J. Food Compos. Anal..

[37] Tironi V.A., Anon M.C. (2010). Amaranth proteins as a source of antioxidant peptides: Effect of proteolysis. Food Res. Int..

[38] Ren Y., Wu H., Li X., Lai F., Xiao X. (2014). Purification and characterization of high antioxidant peptides from duck egg white protein hydrolysates. Biochem. Biophys. Res. Commun..

[39] Esteve C., Marina M.L., García M.C. (2015). Novel strategy for the revalorization of olive (*Olea europaea*) residues based on the extraction of bioactive peptides. Food Chem..

[40] Ngoh Y.Y., Gan C.Y. (2016). Enzyme-assisted extraction and identification of antioxidant and α-amylase inhibitory peptides from Pinto beans (*Phaseolus vulgaris* cv. Pinto). Food Chem..

[41] Chen M., Li B. (2012). The effect of molecular weights on the survivability of casein-derived antioxidant peptides after the simulated gastrointestinal digestion. Innov. Food Sci. Emerg. Technol..

[42] Lin S.Y., Jin Y., Liu M.Y., Yang Y., Zhang M.S., Guo Y., Jones G., Liu J.B., Yin Y.G. (2013). Research on the preparation of antioxidant peptides derived from egg white with assisting of high-intensity pulsed electric field. Food Chem..

[43] Saidi S., Deratani A., Belleville M.P., Amar R.B. (2014). Antioxidant properties of peptide fractions from tuna dark muscle protein by-product hydrolysate produced by membrane fractionation process. Food Res. Int..

[44] Torres-Fuentes C., Contreras M.M., Recio I., Alaiz M., Vioque J. (2015). Identification and characterization of antioxidant peptides from chickpea protein hydrolysates. Food Chem..

[45] Mendis E., Rajapakse N., Byun H.G., Kim S.K. (2005). Investigation of jumbo squid (*Dosidicus gigas*) skin gelatin peptides for their *in vitro* antioxidant effects. Life Sci..

[46] Bougatef A., Nedjar-Arroume N., Manni L., Ravallec R., Barkia A., Guillochon D., Nasri M. (2010). Purification and identification of novel antioxidant peptides from enzymatic hydrolysates of sardinelle (*Sardinella aurita*) by-products proteins. Food Chem..

[47] He R., Ju X., Yuan J., Wang L., Girgih A.T., Aluko R.E. (2012). Antioxidant activities of rapeseed peptides produced by solid state fermentation. Food Res. Int..

[48] Chen H., Zhao M., Lin L., Wang J., Sun-Waterhouse D., Dong Y., Zhuang M., Su G. (2015). Identification of antioxidant peptides from defatted walnut meal hydrolysate with potential for improving learning and memory. Food Res. Int..

[49] Eftekharzadeh B., Khodagholi F., Abdi A., Maghsoudi N. (2010). Alginate protects NT2 neurons against H_2_O_2_-induced neurotoxicity. Carbohydr. Polym..

[50] Najafian L., Babji A.S. (2015). Isolation, purification and identification of three novel antioxidant peptides from patin (*Pangasius sutchi*) myofibrillar protein hydrolysates. LWT-Food Sci. Technol..

[51] Bamdad F., Ahmed S., Chen L. (2015). Specifically designed peptide structures effectively suppressed oxidative reactions in chemical and cellular systems. J. Funct. Foods.

[52] Canabady-Rochelle L.L.S., Harscoat-Schiavo C., Kessler V., Aymes A., Fournier F., Girardet J.M. (2015). Determination of reducing power and metal chelating ability of antioxidant peptides: Revisited methods. Food Chem..

[53] Farvin K.H.S., Baron C.P., Nielsen N.S., Otte J., Jacobsen C. (2010). Antioxidant activity of yoghurt peptides: Part 2—Characterisation of peptide fractions. Food Chem..

[54] Ren J., Zhao M., Shi J., Wang J., Jiang Y., Cui C., Kakuda Y., Xue S.J. (2008). Purification and identification of antioxidant peptides from grass carp muscle hydrolysates by consecutive chromatography and electrospray ionization-mass spectrometry. Food Chem..

[55] Li Y.W., Li B. (2013). Characterization of structure-antioxidant activity relationship of peptides in free radical systems using QSAR models: Key sequence positions and their amino acid properties. J. Theor. Biol..

[56] Kaur H., Garg A., Raghava G.P. (2007). PEP str: A de ovo method for tertiary structure prediction of small bioactive peptides. Protein Pept. Lett..

[57] Jia Z., Natarajan P., Forte T.M., Bielicki J.K. (2002). Thiol-bearing synthetic peptides retain the antioxidant activity of apolipoproteinA-IMilano. Biochem. Biophys. Res. Commun..

[58] Jiménez-Escrig A., Alaiz M., Vioque J., Rupérez P. (2010). Health-promoting activities of ultrafiltered okara protein hydrolysates released by *in vitro* gastrointestinal digestion: Identification of active peptide from soybean lipoxygenase. Eur. Food Res Technol..

[59] Hsieh C.C., Hernández-Ledesma B., Jeong H.J., Park J.H., de Lumen B.O. (2010). Complementary roles in cancer prevention: Protease inhibitor makes the cancer preventive peptide lunasin bioavailable. PLoS ONE.

[60] Northfield S.E., Wang C.K., Schroeder C.I., Durek T., Kan M.W., Swdberg J.E. (2014). Disulfide-rich macrocyclic peptides as templates in drug design. Eur. Food Res Technol..

[61] Li Z., Jiang A., Yue T., Wang J., Wang Y., Su J. (2013). Purification and identification of five novel antioxidant peptides from goat milk casein hydrolysates. J. Dairy Sci..

[62] Wegner J.K., Sterling A., Guha R., Bender A., Faulon J.L., Hastings J., O’Boyle N., Overington J., van Vlijmen H., Willighagen E. (2012). Open-source chemistry software and molecular databases broaden the research horizons in drug discovery. Commun. ACM.

[63] Cheng Y., Luo F., Zeng Z., Wen L., Xiao Z. (2015). DFT-based quantitative structure-activity relationship studies for antioxidant peptides. Struct. Chem..

[64] Li-Chan E.C.Y. (2015). Bioactive peptides and protein hydrolysates: Research trends and challenges for application as nutraceuticalsand functional food ingredients. Curr. Opin. Food Sci..

